# Supplementary material for: Resolution of Halogenated Mandelic Acids through Enantiospecific Co-Crystallization with Levetiracetam
Source: Molecules. 2021 Sep 12;26(18):5536. doi: 10.3390/molecules26185536 (PMC8465588; doi:10.3390/molecules26185536)
Supplement: Supplementary file 1 [file molecules-26-05536-s001.zip › molecules-1380809-supplementary.pdf]

molecules

Supplementary Material

Resolution of Halogenated Mandelic Acids through Enantiospecific Co-Crystallization with Levetiracetam

Jie Wang[1] and Yangfeng Peng [1,*]

[1] School of Chemical Engineering, East China University of Science and Technology, Shanghai 200237, China; 3022740094@qq.com
* Correspondence: yfpeng@ecust.edu.cn; Tel.: +86-21-64252345

Table of Contents:

1. Preparation of co-crystal.
2. X-ray powder diffraction analysis (XRPD analysis).
3. Differential scanning calorimetry analysis (DSC analysis).
4. Infrared spectrum analysis (IR analysis).
5. Solid nuclear magnetic resonance analysis (^{13}C NMR analysis).
6. Elemental analysis (EA).

1. Preparation of Co-Crystal

Liquid assisted-grinding method: The 1:1 co-crystal of LEV and (*S*)-2-ClMA was synthesized by solvent-drop grinding of an equimolar amount of LEV and (*S*)-2-ClMA (e.g., 120 mg total amount of LEV and (*S*)-2-ClMA were mixed with 40 μL of methanol), namely LEV-(*S*)-2-ClMA(G) (G represents the liquid assisted-grinding method). The grinding process lasted for one and a half hours until complete conversion to the co-crystal occurred, and it was then allowed to dry at room temperature. The 1:1 co-crystal LEV-(*S*)-3-ClMA(G) and 1:1 co-crystal LEV-(*S*)-4-ClMA(G) were obtained by the same process. The 1:2 co-crystal LEV-(*S*)-4-BrMA(G) and 1:2 co-crystal LEV-(*R*)-4-FMA(G) were synthesized by solvent-drop grinding of LEV and (*S*)-4-BrMA/ (*R*)-4-FMA in the stoichiometric ratio of 1:2. The process is essentially the same as that described for LEV-(*S*)-2-ClMA(G).
Cooling crystallization method: The 1:1 co-crystal of LEV and (*S*)-2-ClMA was synthesized in a vial with an equimolar amount of LEV and (*S*)-2-ClMA in acetonitrile as solvent, namely LEV-(*S*)-2-ClMA(C) (C represents the cooling crystallization method). The vial was hermetically sealed and heated up to 60 °C until complete dissolution. The solution was slowly cooled to room temperature and a co-crystal LEV-(*S*)-2-ClMA(G) was seeded in −15 °C. After two weeks, the system was assumed to have reached equilibrium and co-crystal was obtained. The 1:1 co-crystal LEV-(*S*)-3-ClMA(C) and 1:1 co-crystal LEV-(*S*)-4-ClMA(C) were obtained by the same process. The 1:2 co-crystal LEV-(*S*)-4-BrMA(C) and 1:2 co-crystal LEV-(*R*)-4-FMA(C) were synthesized in a vial with the

stoichiometric ratio of 1:2 of LEV and (*S*)-4-BrMA/(*R*)-4-FMA in acetonitrile. The process is essentially the same as that described for LEV-(*S*)-2-ClMA(C).

2. X-ray powder diffraction analysis (XRPD analysis)

XRPD spectra of co-crystal LEV-(*S*)-2-ClMA and their raw materials are shown in Figure S1(a). LEV has characteristic peaks at 10.29°, 15.20°, 18.67°, 26.93°, and 30.46°, and (*S*)-2-ClMA has characteristic peaks at 6.62° and 10.46°. On the XRPD spectrum of LEV-(*S*)-2-ClMA(G), the characteristic peaks of the two raw materials disappeared completely, and new characteristic peaks appeared at 7.02°, 13.94°, 16.73°, 22.66°, 24.57°, and 27.59°, indicating that LEV-(*S*)-2-ClMA(G) is a new material different from the raw materials. As shown in Figure S1(b), S1(c), S1(d), and S1(e), the diffraction peaks of co-crystal LEV-(*S*)-3-ClMA(G), LEV-(*S*)-4-ClMA(G), LEV-(*S*)-4-BrMA(G), and LEV-(*R*)-4-FMA(G) are different from their raw materials, which indicates that new substances have been formed. Compared with the XRPD spectra of co-crystals obtained by liquid-assisted grinding, the co-crystals obtained by cooling crystallization have the same characteristic peaks, indicating that the co-crystals obtained by the two methods are the same and belong to the same substance.

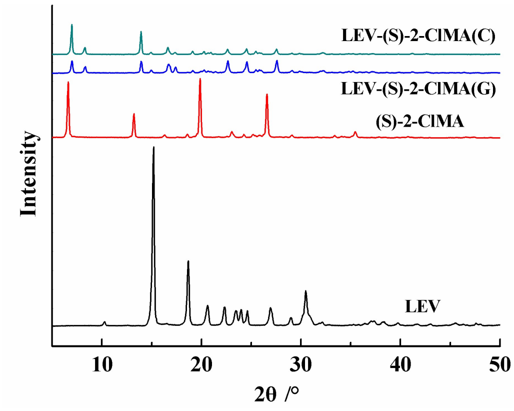

(a) LEV, (*S*)-2-ClMA, and co-crystal

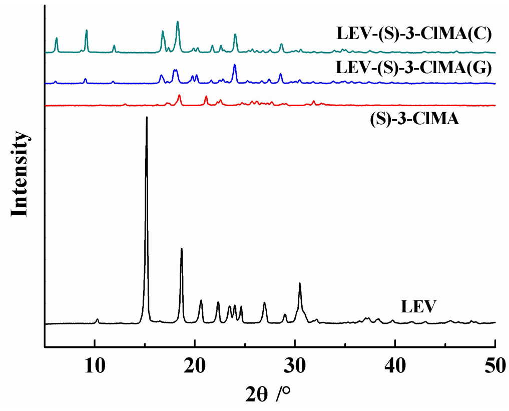

(b) LEV, (*S*)-3-ClMA, and co-crystal

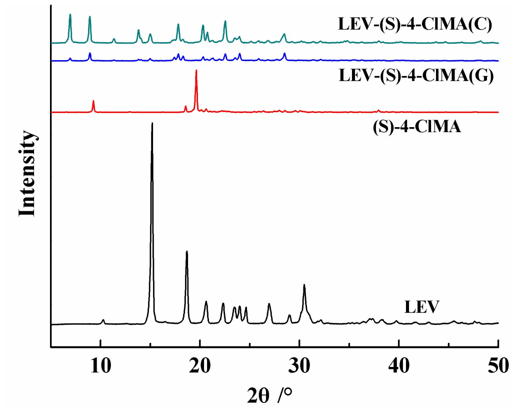

(c) LEV, (*S*)-4-ClMA, and co-crystal

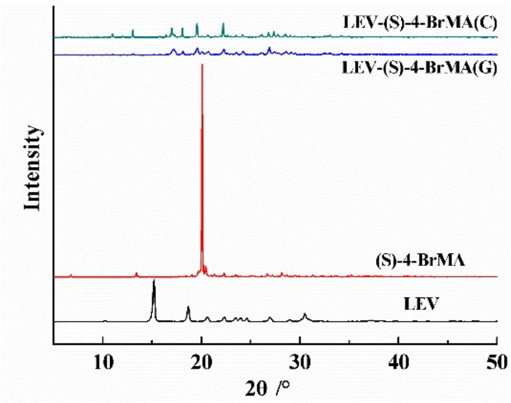

(d) LEV, (*S*)-4-BrMA, and co-crystal

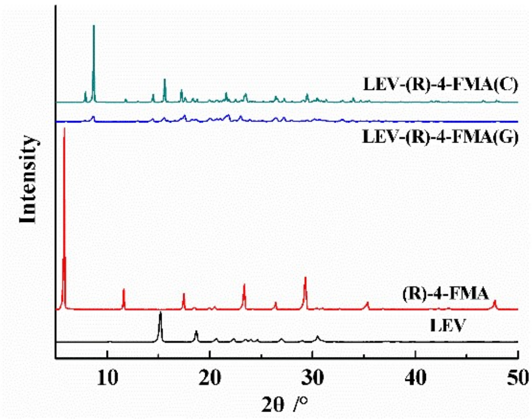

(e) LEV, (*R*)-4-FMA, and co-crystal

Figure S1. XRPD spectra of LEV, halogenated mandelic acids, and co-crystals.

3. Differential scanning calorimetry analysis (DSC analysis)

Thermodynamic analysis is an important means to characterize the generation of new substances. Table 1 shows the thermodynamic properties of LEV, halogenated mandelic acids, and five co-crystals, including the melting point and heat of fusion. It shows that the melting point and heat of fusion of the five co-crystals are different from those of the two raw materials. Figure S2 shows DSC curves of five co-crystals, and T_{max} values are marked on the curves. In five systems, the endothermic peaks of raw materials disappeared completely in DSC curves of co-crystals obtained by liquid-assisted grinding, and new endothermic peaks appeared at positions different from those of the two raw materials, and their melting points were lower than those of the two raw materials. It can be proved that grinding product is a new substance different from raw materials, which is a thermodynamic sign of co-crystal formation, and the co-crystals show lower thermal stability than the raw materials.

Table S1. Thermodynamic properties of LEV, halogenated mandelic acids, and co-crystals.

	**Melting point /℃**	**Heat of fusion/J . g^{-1}**
LEV	117.7	166.28
(*S*)-2-ClMA	119.5	127.57
LEV-(*S*)-2-ClMA(G)	97.4	69.66
(*S*)-3-ClMA	106.8	114.02
LEV-(*S*)-3-ClMA(G)	84.7	93.66
(*S*)-4-ClMA	118.4	69.76
LEV-(*S*)-4-ClMA(G)	73.6	59.17
(*S*)-4-BrMA	130.4	104.00
LEV-(*S*)-4-BrMA(G)	90.1	35.55
(*R*)-4-FMA	146.5	92.03
LEV-(*R*)-4-FMA(G)	69.4	65.09

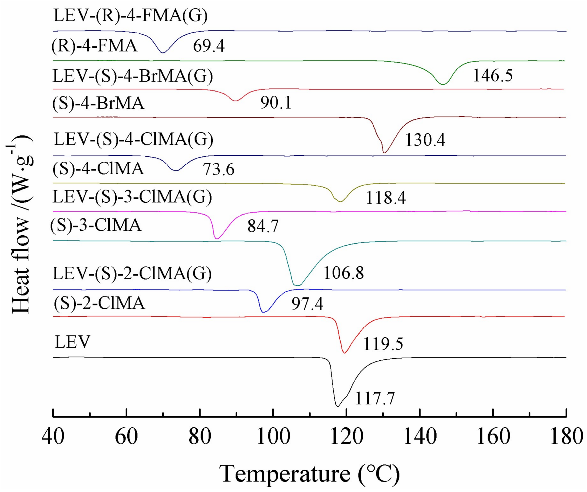

Figure S2. DSC curves of LEV, halogenated mandelic acids, and co-crystals.

4. Infrared spectrum analysis (IR analysis)

Figure S4(a) is the IR spectrum of LEV, (*S*)-2-ClMA, and co-crystal LEV-(*S*)-2-ClMA(G). As shown in the figure, the absorption peaks of LEV at 3361.8 cm^{-1} and 3194.0 cm^{-1} belong to the stretching vibration of two –NH of amide group, and in the IR spectrum of LEV-(*S*)-2-ClMA(G), the two absorption peaks are blue shifted to 3434.7 cm^{-1} and 3271.4 cm^{-1}, respectively. The absorption peaks of LEV at 1678.3 cm^{-1} and 1650.8 cm^{-1} are attributed to the stretching vibration of C1═O and C6═O, and both of them are redshifted to 1493.2 cm^{-1}. The absorption peaks of (*S*)-2-ClMA at 3380.3 cm^{-1} and 1750.9 cm^{-1} belong to the stretching vibration of the hydroxyl and carboxyl group, respectively, and are redshifted to 3332.4 cm^{-1} and 1660.5 cm^{-1}, respectively, in LEV-(*S*)-2-ClMA(G). As shown in Figure S4(b), S4(c), S4(d), and S4(e), the position change of the functional group absorption peak also occurs in co-crystal LEV-(*S*)-3-ClMA(G), LEV-(*S*)-4-ClMA(G), LEV-(*S*)-4-BrMA(G), and LEV-(*R*)-4-FMA(G). From the results of IR analysis, it can be found that the migration phenomenon of these groups' stretching vibration peaks occurs, which is due to the formation of hydrogen bonds between functional groups and the change of chemical environment of groups during the co-crystal formation process, and the formation of co-crystal can be judged.

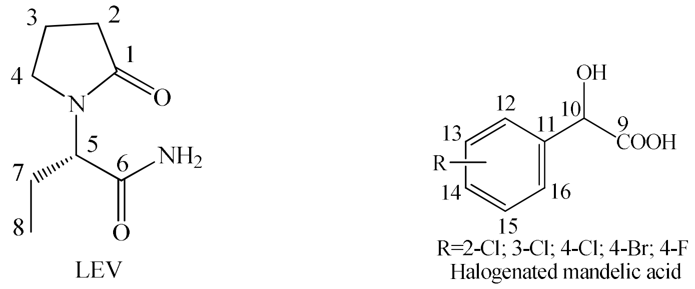

Figure S3. Carbon number of LEV and halogenated mandelic acid.

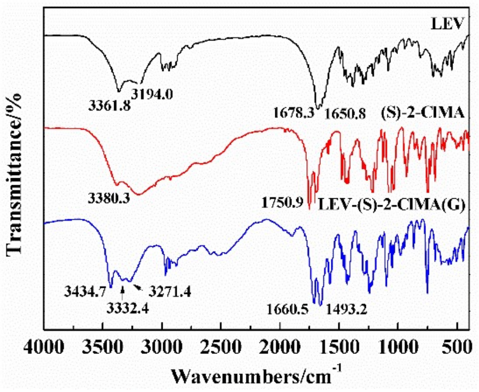

(a) LEV, (S)-2-ClMA, and co-crystal

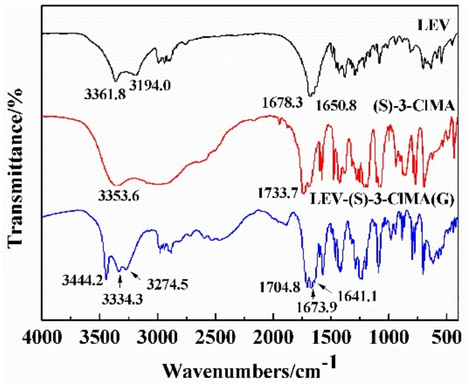

(b) LEV, (S)-3-ClMA, and co-crystal

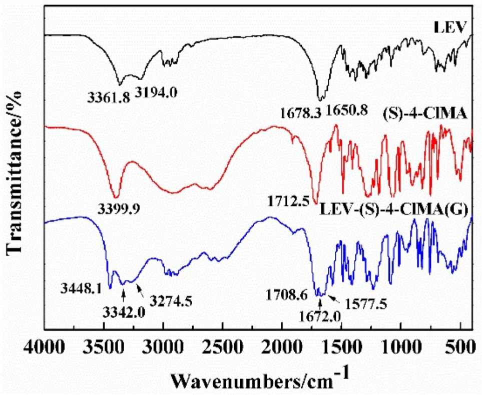

(c) LEV, (S)-4-ClMA, and co-crystal

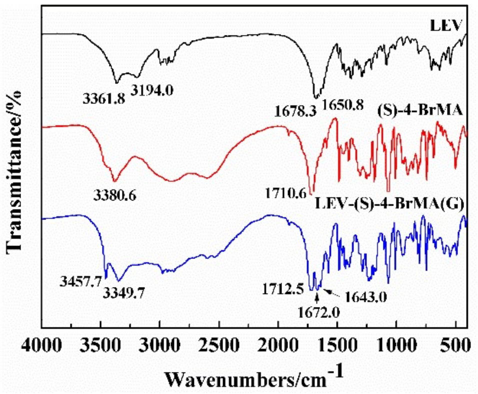

(d) LEV, (S)-4-BrMA, and co-crystal

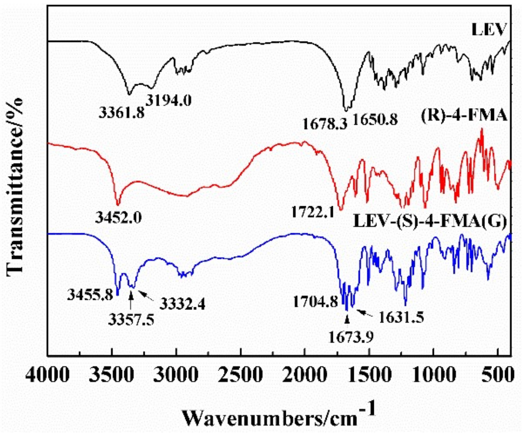

(e) LEV, (R)-4-FMA, and co-crystal

Figure S4. IR spectra of LEV, halogenated mandelic acids, and co-crystals.

5. Solid nuclear magnetic resonance analysis (^{13}C NMR analysis)

Table S2. ^{13}C NMR data of LEV, halogenated mandelic acids, and co-crystals.

Chemical shift/ppm	C1	C6	C9	C10
LEV	173.95	177.9	-	-
(*S*)-2-ClMA	-	-	178.77	68.87
LEV-(*S*)-2-ClMA(G)	176.64	179.65	179.65	73.01
(*S*)-3-ClMA	-	-	175.48	72.76
LEV-(*S*)-3-ClMA(G)	175.61	178.50	178.50	73.53

(*S*)-4-ClMA	-	-	175.54	70.77
LEV-(*S*)-4-ClMA(G)	175.35	179.06	177.90	73.29
(*S*)-4-BrMA	-	-	176.64	72.45
LEV-(*S*)-4-BrMA(G)	175.17	178.64	177.05	71.52
(*R*)-4-FMA	-	-	175.05	71.87
LEV-(*R*)-4-FMA(G)	172.44	178.76	172.44	72.96

^{13}C NMR data of raw materials LEV, halogenated mandelic acids, and five co-crystals are shown in Table 2, and the chemical shifts of C1, C6, C9, and C10 all migrate, indicating that hydrogen bonds are formed between functional groups during co-crystal formation, which is consistent with the chemical shift migration of no more than 5 ppm during co-crystal formation, further proving the formation of co-crystal. In ^{13}C NMR spectra of co-crystal LEV-(*S*)-2-ClMA(G), LEV-(*S*)-3-ClMA(G), and LEV-(*S*)-4-ClMA(G), the peaks of carbon atoms did not split, indicating that the co-crystals formed are 1:1 co-crystals. In ^{13}C NMR spectra of LEV-(*S*)-4-BrMA(G), and LEV-(*R*)-4-FMA(G), the peaks of carbon atoms split, indicating that the co-crystals formed are 1:2 co-crystals.

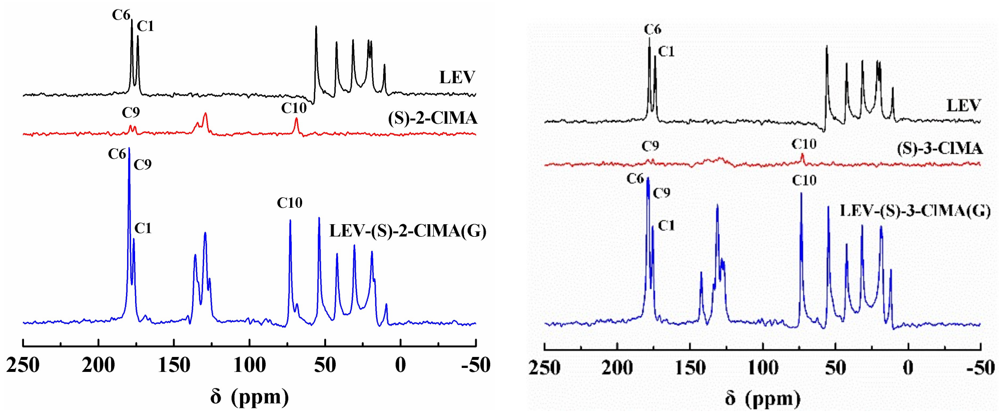

(a) LEV, (S)-2-ClMA, and co-crystal (b) LEV, (S)-3-ClMA, and co-crystal

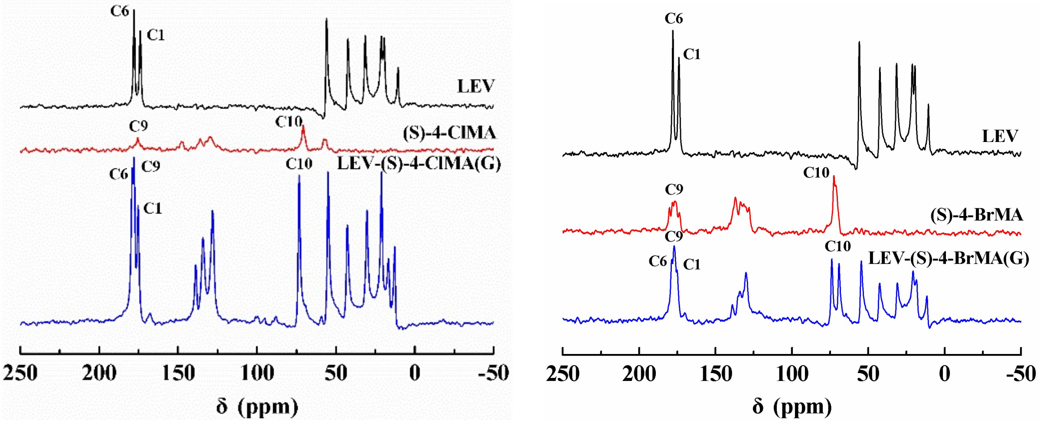

(c) LEV, (S)-4-ClMA, and co-crystal (d) LEV, (S)-4-BrMA, and co-crystal

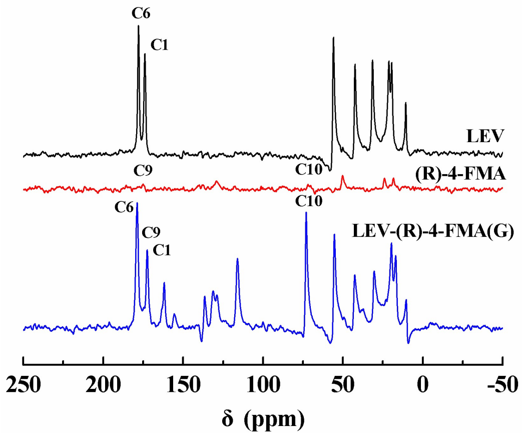

(e) LEV, (R)-4-FMA, and co-crystal

Figure S5. ^{13}C NMR of LEV, halogenated mandelic acids, and co-crystals.

6. Elemental analysis (EA)

Five co-crystals obtained by cooling crystallization were analyzed and tested, and the percentage contents of C, H, and N elements were obtained. The theoretical contents of C, H, and N elements were obtained by calculating the five co-crystals with LEV:(*S*)-2-ClMA=1:1, LEV:(*S*)-3-ClMA=1:1, LEV:(*S*)-4-ClMA=1:1, LEV:(*S*)-4-BrMA=1:2, and LEV:(*R*)-4-FMA=1:2. The measured and theoretical values of C, H, and N elements are shown in Table S3, which shows that the measured and theoretical values are basically consistent, and the relative errors are all within 5%.

Table S3. Elemental analysis results of five co-crystals.

	C/%		H/%		N/%	
	**Theoretical value**	**Measured value**	**Theoretical value**	**Measured value**	**Theoretical value**	**Measured value**
LEV-(*S*)-2-ClMA(C)	53.89	54.10	5.93	5.92	7.85	7.86
LEV-(*S*)-3-ClMA(C)	53.89	53.98	5.93	5.88	7.85	7.81
LEV-(*S*)-4-ClMA(C)	53.89	53.98	5.93	5.91	7.85	7.93
LEV-(*S*)-4-BrMA(C)	45.57	45.68	4.43	4.36	4.43	4.26
LEV-(*R*)-4-FMA(C)	56.42	56.30	5.48	5.52	5.48	5.30
